# Supplementary material for: Do subterranean mammals use the Earth’s magnetic field as a heading indicator to dig straight tunnels?
Source: PeerJ. 2018 Oct 31;6:e5819. doi: 10.7717/peerj.5819 (PMC6215444; doi:10.7717/peerj.5819)
Supplement: Supplemental Information 1 [file peerj-06-5819-s001.docx]

**Supplementary information**

**Do subterranean mammals use the Earth’s magnetic field as a heading indicator to dig straight tunnels?**

Sandra Malewski, Sabine Begall, Cristian E. Schleich, Carlos D. Antenucci, Hynek Burda


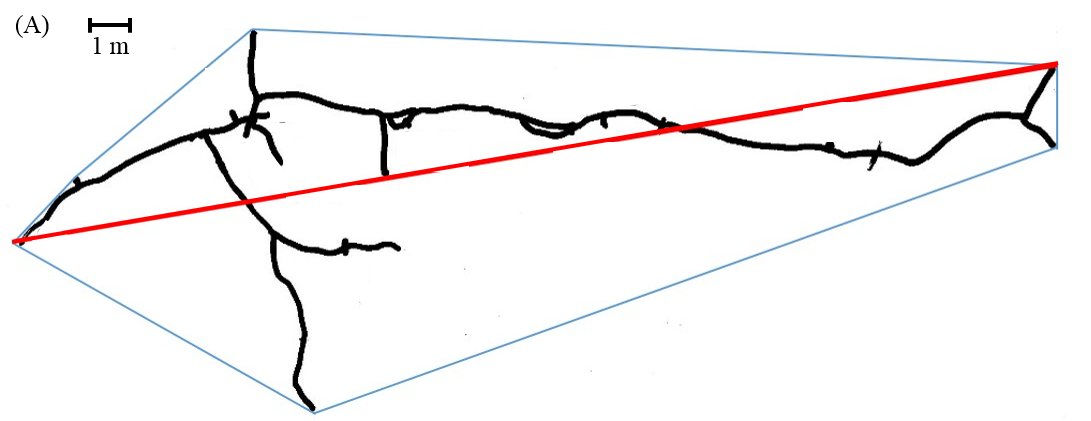


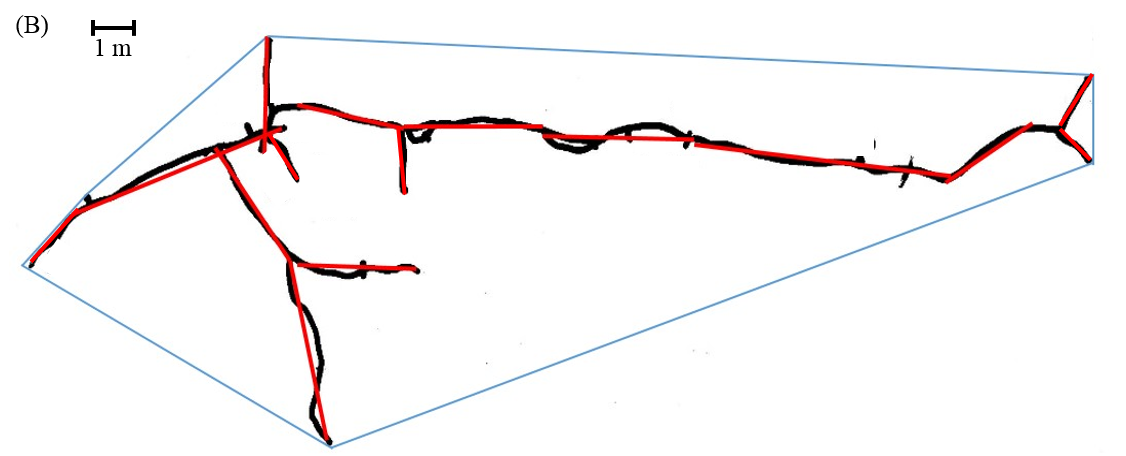


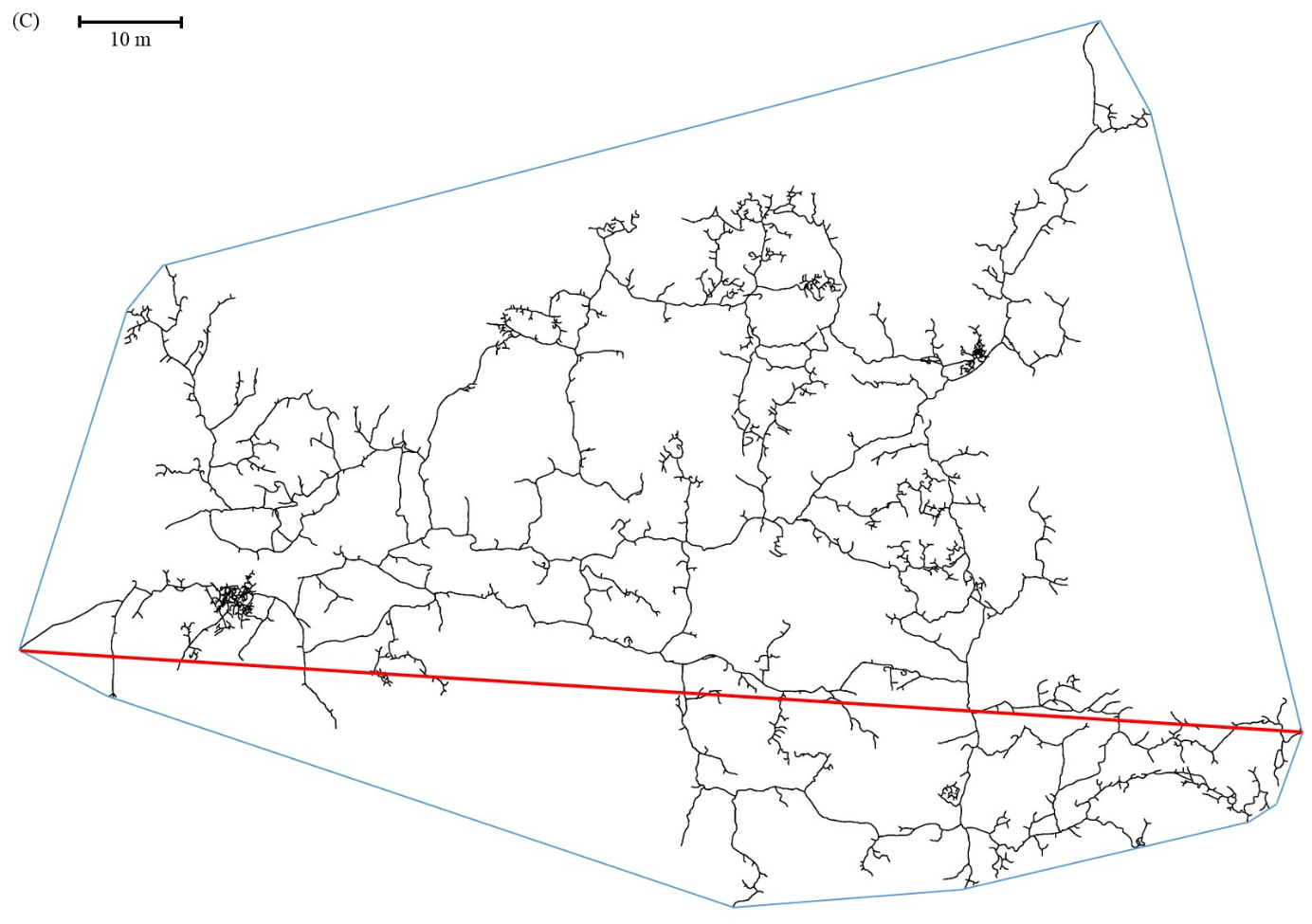


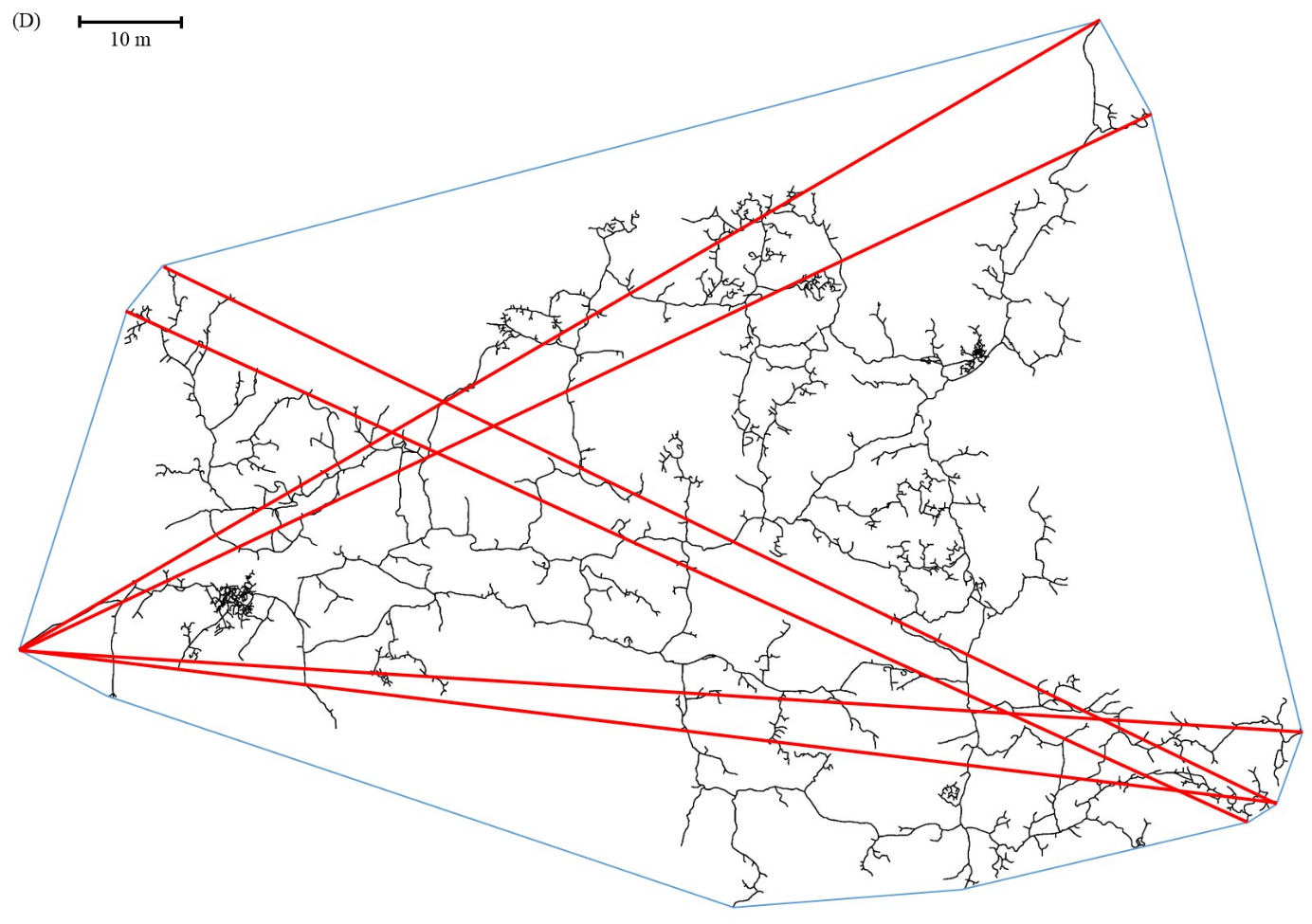


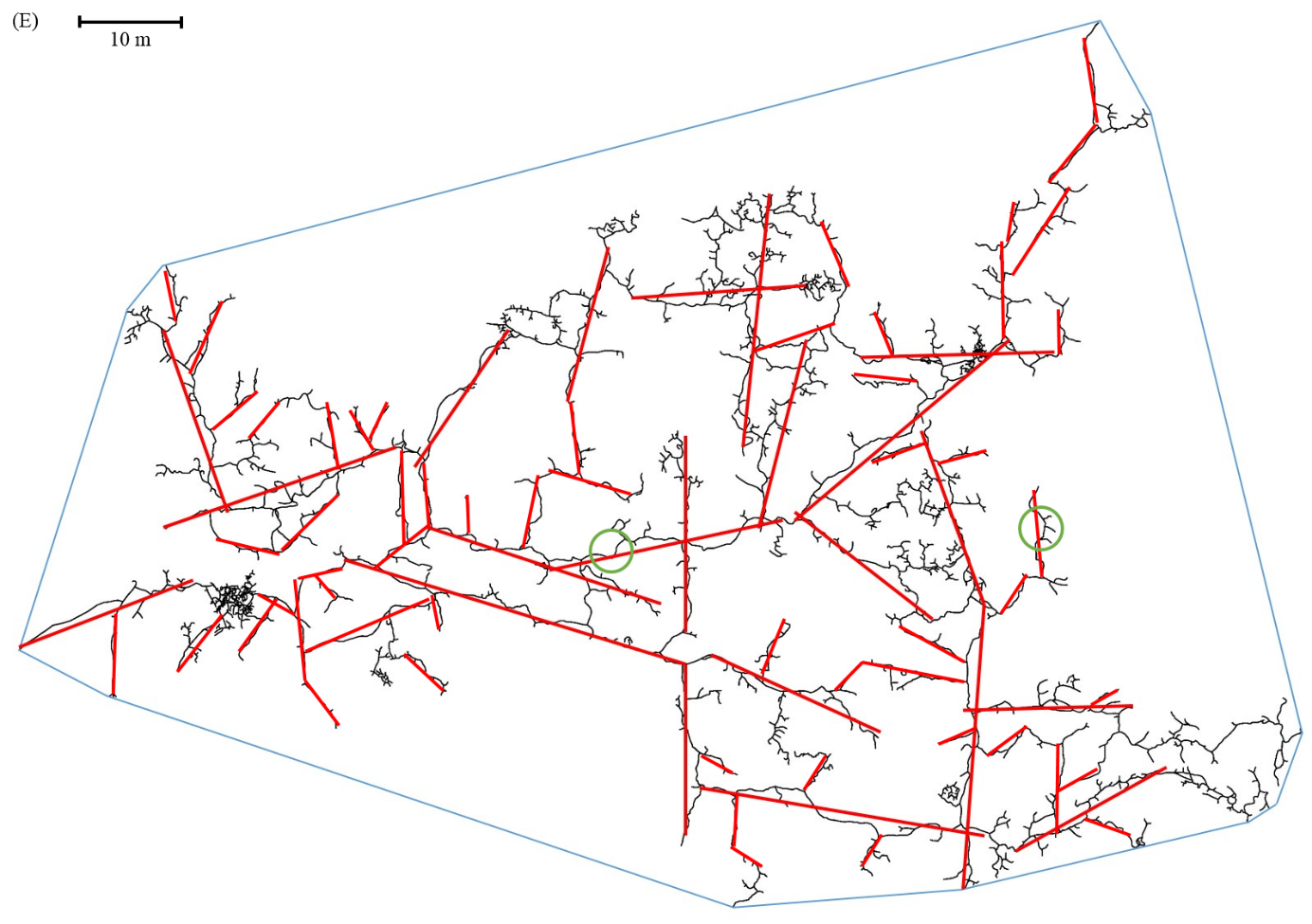


Figure S1: Exemplary demonstration of the polygon (A, C, D) and long tunnel method’s (B, E) application. In (A) and (B) a burrow system of a solitary silvery mole-rat (*H. argenteocinereus*) and in (C), (D), and (E) a burrow system of a family of social Ansell’s mole-rats (*F. anselli*) is shown. A convex polygon surrounds each burrow system (marked in blue), respectively. In (A) and (C), the longest distance within the polygon, representing its main orientation, is drawn in (red line). In (D), six diagonals of similar lengths are drawn into the polygon, pointing out the problem of determining only one main axis by using the polygon method. In (E), straight tunnels, including small turns/curves (exemplarily marked by green circles) that could be interpreted e. g. as the animal’s avoidance of an obstacle, are marked by means of red lines.
